# Book Notices

**Published:** 1884-02-20

**Authors:** 


### *BOOK NOTICES.*

*Hand-Book of Eclampsia*, or Notes on Cases of Puerperal Convulsions, comprising all the cases which have occurred during the present century within a radius of several miles around Avondale, Chester Co., Penn., so far as can be ascertained, by E. Mincher, M.D., I. H. Stubbs, M.D., E. Thompson, M.D., R. B. Ewing, M.D., S. Stebbins, M.D. Philadelphia, 1883. F. A. Davis, 1217 Elbert street.

This is a little work of sixty-eight pages. The cases reported are interesting and suggestive, the drift of which is to show that the *free* use of the lancet is the remedy *par excellence* for Eclampsia. It is therein affirmed that "The proximate cause of the trouble is too much blood in the brain, heart and lungs. Remove the blood and the effect will cease."

*The Roller Bandage*, by Wm. Barton Hopkins, M.D., Surgeon to the Out Departments of Pennsylvania Episcopal and University Hospitals, Assistant Demonstrator of Surgery in the University of Pennsylvania, Fellow of the College of Physicians, of Philadelphia, etc., with 73 illustrations. Philadelphia: J. B. Lippincott & Co. 1883.

This is an interesting and eminently practical little work of ninety-five pages on the various methods of applying the roller bandage. It is illustrated from life and contains a series of definitions and rules for bandaging, with very brief and plain descriptions for applying the bandages, their uses, &c.

